# The use of a uPAR-targeted probe for photothermal cancer therapy prolongs survival in a xenograft mouse model of glioblastoma

**DOI:** 10.18632/oncotarget.28013

**Published:** 2021-07-06

**Authors:** Marina Simón, Jesper Tranekjær Jørgensen, Karina Juhl, Andreas Kjaer

**Affiliations:** ^1^Department of Clinical Physiology, Nuclear Medicine & PET and Cluster for Molecular Imaging, Department of Biomedical Sciences, Rigshospitalet and University of Copenhagen, Copenhagen, Denmark

**Keywords:** photothermal therapy (PTT), indocyanine green (ICG), urokinase plasminogen activator receptor (uPAR), cancer, hyperthermia

## Abstract

The development of tumor-targeted probes that can efficiently reach cancerous tissue is an important focus of preclinical research. Photothermal cancer therapy (PTT) relies on light-absorbing molecules, which are directed towards tumor tissue and irradiated with an external source of light. This light is transformed into heat, causing localized hyperthermia and tumor death. The fluorescent probe indocyanine green (ICG) is already used as an imaging agent both preclinically and in clinical settings, but its use for PTT is yet to be fully exploited due to its short retention time and non-specific tumor targeting. Therefore, increasing ICG tumor uptake is necessary to improve treatment outcome. The urokinase-type plasminogen activator receptor, uPAR, is overexpressed in multiple tumor types. ICG-Glu-Glu-AE105, consisting of the uPAR-targeting peptide AE105 conjugated to ICG, has shown great potential for fluorescence-guided surgery. In this study, ICG-Glu-Glu-AE105 was evaluated as photothermal agent in a subcutaneous mouse model of human glioblastoma. We observed that the photothermal abilities of ICG-Glu-Glu-AE105 triggered high temperatures in the tumor during PTT, leading to tumor death and prolonged survival. This confirms the potential of ICG-Glu-Glu-AE105 as photothermal agent and indicates that it could be used as an add-on to the application of the probe for fluorescence-guided surgery.

## INTRODUCTION

The development of new and more efficient ways to induce localized tumor death without damaging healthy tissue is still a need in cancer treatment and management. For this, photothermal cancer therapy (PTT) holds great potential; this therapy relies on photoabsorbing molecules, which are directed towards tumor tissue, and are able to transform the near-infrared (NIR) light they are irradiated with into heat, causing highly localized tumor death through hyperthermia [1, 2]. At present, the most efficient photothermal agents are metallic nanoparticles, which present the disadvantage that they are non-biodegradable, and therefore remain in the body for long periods of time [3]. Currently, other molecules with photothermal abilities are being investigated. This includes indocyanine green (ICG), a fluorophore that is FDA approved for determining cardiac output, liver blood flow and hepatic function as well as ophthalmic angiography [4, 5]. Additionally, it is currently also being tested in clinical trials as a probe for fluorescence-guided surgery [6]. The properties of ICG have been studied preclinically, but a short circulation and retention time in tumor tissue are considerable limitations to the use of ICG as a photothermal agent [7–9]. As a higher degree of tumor retention of ICG would improve treatment outcome, some studies have focused on achieving a more specific tumor accumulation of ICG by using an ICG-loaded nanocarrier or by linking the fluorophore to a molecule targeting tumor components [10–12].

Many tumors express high levels of the urokinase-type plasminogen activator receptor (uPAR). When uPA binds to uPAR, a proteolytic cascade is initiated and leads to the destruction of extracellular matrix components. Apart from regulating proteolysis of uPA, uPAR has been studied as a promising therapeutic target in cancer due to its ability to activate multiple intracellular signaling pathways leading to cell adhesion, proliferation and migration. uPAR also plays a role in regulating cancer cell dormancy and angiogenesis [13, 14]. Importantly, overexpression of uPAR is associated with a more invasive and aggressive cancer progression [15].

In this study, we investigated the potential of ICG-Glu-Glu-AE105 for image-guided photothermal cancer therapy in a subcutaneous xenograft mouse model of human glioblastoma. AE105 is a small peptide that acts as a uPAR agonist, and together with ICG, constitutes a fluorescent probe that targets uPAR-expressing tumor cells and at the same time can be followed throughout the body with fluorescence imaging techniques. The imaging properties of ICG-Glu-Glu-AE105 have already been described in preclinical studies, and have also shown potential for intra-operative optical imaging [16]. Mice bearing human glioblastoma U-87 MG tumors have previously shown tumor accumulation of AE105 [16–18]. Thus, we hypothesized that ICG-Glu-Glu-AE105 could potentially be an effective photothermal agent, and ICG-Glu-Glu-AE105-based PTT could therefore be used as an adjunct to image-guided surgery with the same compound.

## RESULTS

### Photothermal abilities of ICG-Glu-Glu-AE105 *in vitro*

**Figure 1:**
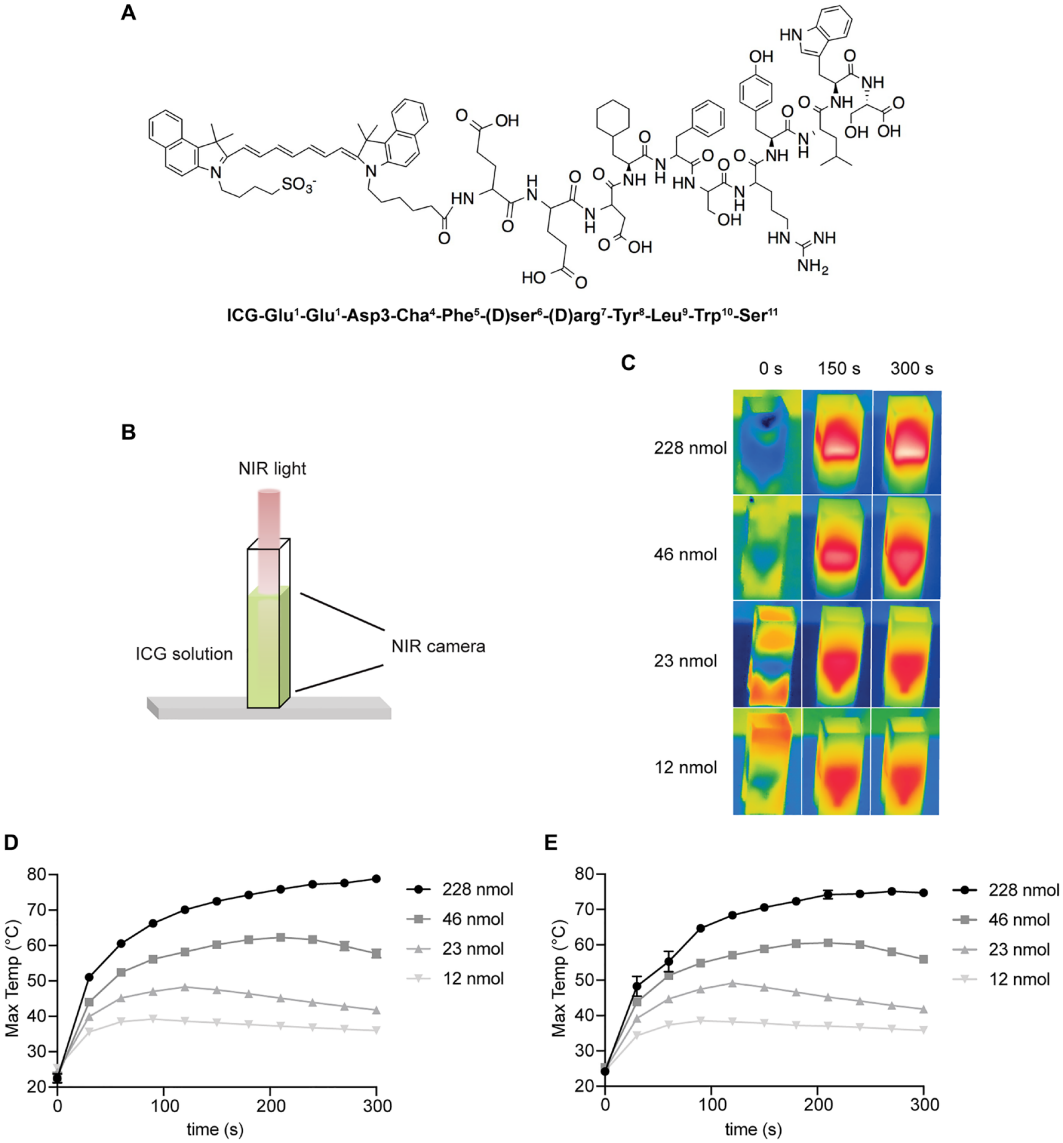
*In vitro* heating of ICG-Glu-Glu-AE105 and unconjugated ICG solutions. (**A**) ICG-Glu-Glu-AE105 structure. (**B**) Graphic representation of the NIR laser pointing towards the cuvette containing the solution. The NIR camera takes pictures from the front of the cuvette. (**C**) Representative images from the FLIR camera showing the distribution of heat in the cuvette at different concentrations of ICG in an ICG-Glu-Glu-AE105 solution. (**D**) Temperature increase during irradiation in the ICG-Glu-Glu-AE105 solution. (**E**) Temperature increase of the ICG solution. Laser intensity of 2 W/cm^2^ for five minutes and *n* = 3. Data shown is mean ± SEM. (standard error of the mean).

In order to test the ability of ICG-Glu-Glu-AE105 (Figure 1A) to heat under NIR light, different concentrations of the compound in a 1-mL solution were placed in a plastic cuvette under a laser beam, at an intensity of 2 W/cm^2^ (Figure 1B). The samples were then irradiated for five minutes and the maximum temperatures recorded with a FLIR (forward-looking infrared) camera (Figure 1C and 1D).

Additionally, the same molar concentrations of ICG in its non-conjugated form were also irradiated in solution (i.e., equal number of ICG moles for both ICG-Glu-Glu-AE105 and unconjugated ICG, Figure 1E), in order to confirm that the conjugation process did not hinder the heat generation abilities of ICG. As it can be observed in Figures 1D and 1E, ICG-Glu-Glu-AE105 and ICG alone followed similar heating patterns, and a temperature of around 80°C was reached for the highest concentration tested (228 nmol). However, it was observed that the heat was mainly localized at the top of the sample and not evenly distributed throughout the solution (Figure 1C). This phenomenon has previously been described by Hogan et al., who showed that when the solution is too dense, photons cannot penetrate deeper into the solution and the light interacts mainly at the surface of the sample [19]. As for a concentration of 46 nmol, heat generation followed a slower pace but maximum temperatures of up to 60°C were reached for both conjugated and unconjugated forms, and the heat distribution was more homogeneous. Finally, lower concentrations of ICG such as 23 and 12 nmol showed earlier peaks and lower maximum temperatures (just below 50°C for 23 nmol and around 38°C for 12 nmol). An important detail observed was the temperature decrease after reaching a certain peak, most likely due to the irreversible degradation of ICG, which impedes heat generation [20].

### uPAR expression in U-87 MG.luc2 cells and ICG-Glu-Glu-AE105 accumulation *in vivo*

**Figure 2:**
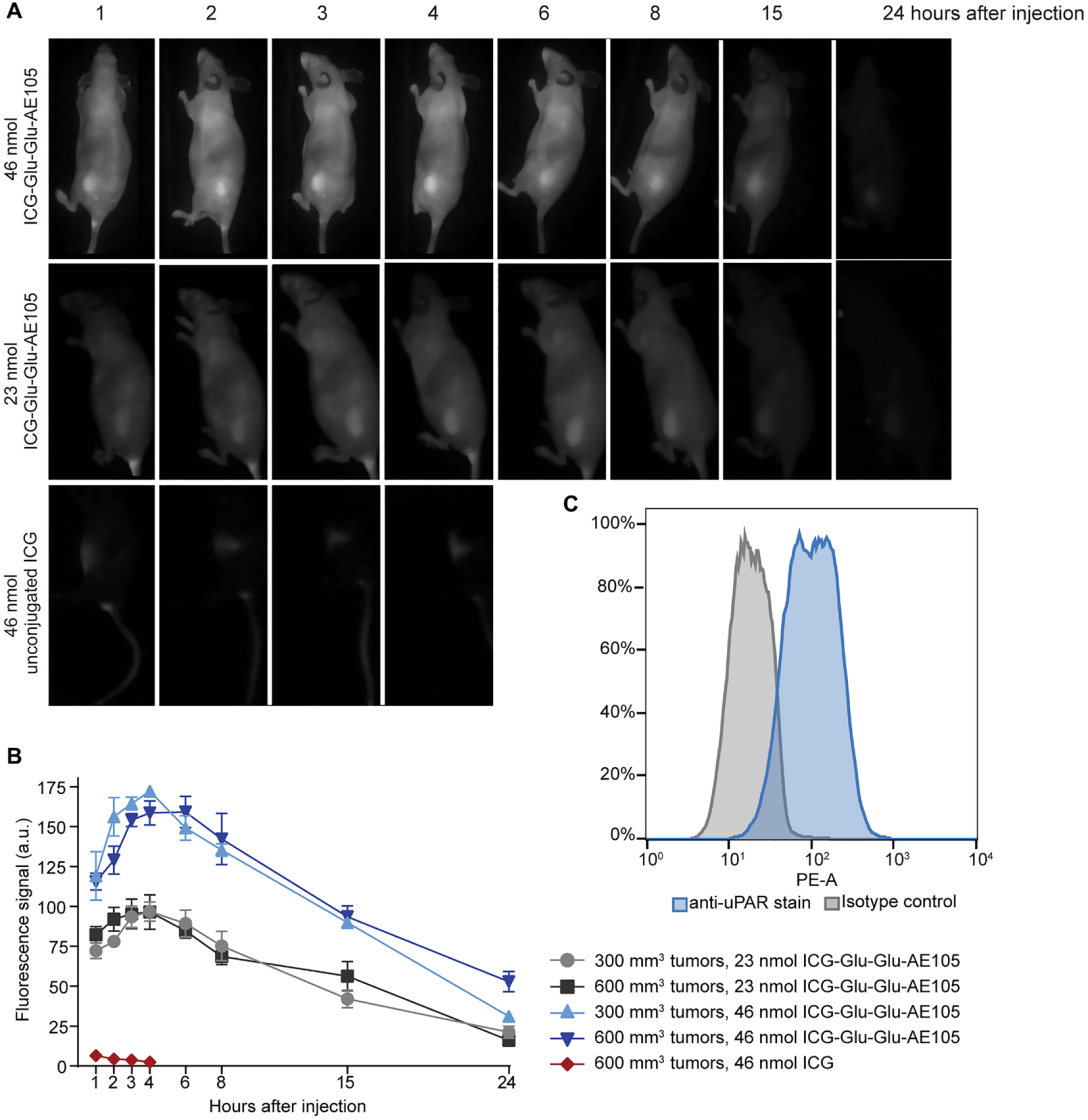
Accumulation of ICG-Glu-Glu-AE105 and unconjugated ICG in subcutaneous U-87 MG.luc2 tumors and uPAR expression *in vitro*. (**A**) Representative images obtained with the Fluobeam^®^800 NIR-camera of ICG-Glu-Glu-AE105 (in 300 mm^3^ tumors) and ICG (in 600 mm^3^ tumors) accumulation at different time points. ICG-Glu-Glu-AE105 uptake in 600m^3^ tumors followed the same pattern as in 300 mm^3^ tumors. (**B**) Fluorescence signal emitted by the ICG molecules in either ICG-Glu-Glu-AE105 or unconjugated ICG throughout time, at different concentrations and tumor sizes. Data shown is mean ± SEM. (**C**) uPAR expression *in vitro* in U-87 MG.luc2 cells studied by flow cytometry. Isotype control is represented in gray, and the sample stained with PE anti-uPAR antibody in blue. 81% of the stained cells were positive for uPAR.

After confirming the ability of ICG-Glu-Glu-AE105 to heat *in vitro,* we proceeded to test the accumulation of the compound in a subcutaneous xenograft mouse model of glioblastoma, using U-87 MG.luc2 cells. First, the expression of uPAR in the U-87 MG.luc2 cells was confirmed by flow cytometry. As shown in Figure 2C, there was a clear shift in fluorescence between the isotype control and the uPAR-stained sample, which underlines that U-87 MG.luc2 cells express uPAR (81% of the cells in the analysis were positive for uPAR).

Next, mice bearing 300 or 600 mm^3^ U-87MG.luc2 tumors were divided into groups (*n* = 3–5 per group) and injected with either 46 or 23 nmol of ICG-Glu-Glu-AE105 in 0.2 mL intravenously. To quantify the accumulation of the ICG-linked agent in the tumor at different time points, the animals were scanned with the Fluobeam^®^800 NIR-camera. In Figure 2A, it is possible to observe that the accumulation of ICG-Glu-Glu-AE105 was highly localized in the tumor, and it reached a peak at around four hours after injection (Figure 2B). As expected, a higher concentration of ICG-Glu-Glu-AE105 resulted in higher levels of accumulation. Interestingly, there were no size-dependent differences in ICG-Glu-Glu-AE105 accumulation for the two tumor volumes included in the study. For future reference, 300 mm^3^ was the tumor size chosen for the subsequent *in vivo* studies.

In order to confirm the tumor specific uptake of ICG-Glu-Glu-AE105, unconjugated ICG (46 nmol) was also studied in the tumors at different timepoints. In this group of mice, no significant tumor uptake was observed. Overall, this confirmed that the AE105 peptide allows for a specific accumulation of the probe in the tumor and that much higher concentrations of unconjugated ICG would be needed to obtain a significant uptake.

### Testing the ability of ICG-Glu-Glu-AE105 to ablate tumors *in vivo*

After deciding on dose (46 nmol) and treatment timepoint (four hours after injection of the probe), different groups of mice were set up in order to test the feasibility of ICG-Glu-Glu-AE105 as a photothermal agent, capable of specifically ablating tumors *in vivo*.

**Figure 3:**
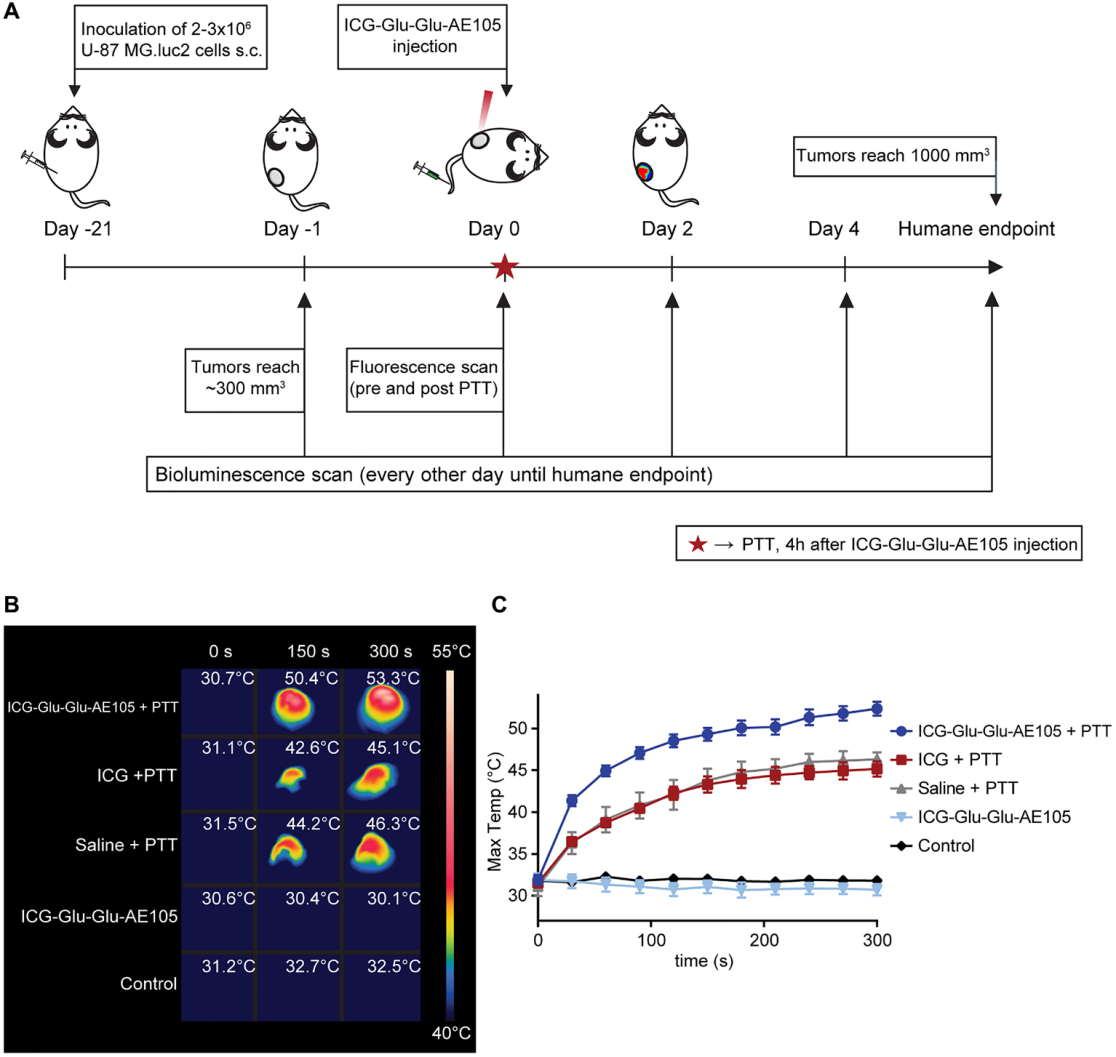
ICG-Glu-Glu-AE105 as photothermal agent *in vivo*. (**A**) Depicts the study timeline. Animals underwent a bioluminescence baseline scan one day before PTT, when tumors reached around 300 mm^3^, and were divided into groups. On day 0, animals were injected with ICG-Glu-Glu-AE105, ICG, or saline four hours before PTT. Animals bearing ICG-Glu-Glu-AE105 were also fluorescence-scanned before and after PTT. Afterwards, animals were scanned for bioluminescence every other day until tumors reached ~1000 mm^3^. (**B**) Representative FLIR images from the different groups (ICG-Glu-Glu-AE105 + PTT; *n* = 6, ICG + PTT; *n* = 4, Saline + PTT; *n* = 4, ICG-Glu-Glu-AE105; *n* = 5 and Control; *n* = 4) during laser irradiation at 2 W/cm^2^. (**C**) Temperatures reached on the tumor surface at different timepoints for the different groups during PTT. Data shown is mean ± SEM.

For this, mice were inoculated subcutaneously (s.c.) with U-87 MG.luc2 cells. The bioluminescent signal, detected when the reactive luciferin was injected and oxidized by the enzyme luciferase, served as a way to determine treatment effect and follow tumor growth. When tumors reached ~300 mm^3^, five groups were set up; ICG-Glu-Glu-AE105 + PTT group (*n* = 6, ICG-Glu-Glu-AE105 injection and laser treatment), ICG + PTT group (*n* = 4, unconjugated ICG injection and laser treatment), saline + PTT group (*n* = 4, saline injection and laser treatment), ICG-Glu-Glu-AE105 group (*n* = 5, ICG-Glu-Glu-AE105 injection but no laser treatment) and a control group (*n* = 4, no treatment). The timeline for the experiments is shown in Figure 3A. First, all mice underwent a baseline bioluminescence scan (day -1), and the following day (day 0) they were injected with either ICG-Glu-Glu-AE105, ICG alone or saline. Approximately four hours later, animals receiving PTT were irradiated for five minutes with NIR light at 2 W/cm^2^, and the temperatures on tumor surface were measured with a FLIR camera. Non-irradiated mice (ICG-Glu-Glu-AE105 and control groups) were placed on the treatment platform for five minutes with the laser turned off. Animals injected with ICG-Glu-Glu-AE105 were imaged with the Fluobeam^®^800 NIR-camera before and after PTT or sham treatment. Additionally, all mice underwent bioluminescence imaging post-treatment, followed by a scan every other day after treatment until the tumors reached 1,000 mm^3^ (humane endpoint).

When tumors from the ICG-Glu-Glu-AE105 + PTT group were irradiated, surface temperatures increased at a quick pace and after five minutes the highest temperature reached was around 52°C, compared to around 45°C for ICG + PTT and saline + PTT groups, which most likely reflects unspecific heating caused by the laser per se (Figure 3B and 3C). The significantly higher temperatures reached by the ICG-Glu-Glu-AE105 + PTT group compared to the unconjugated ICG + PTT group are an effect of the high specificity of ICG-Glu-Glu-AE105 in the tumor, resulting in significant tumor death through localized heating. As expected, non-treated ICG-Glu-Glu-AE105 and control tumors did not show any change in temperature over time.

**Figure 4:**
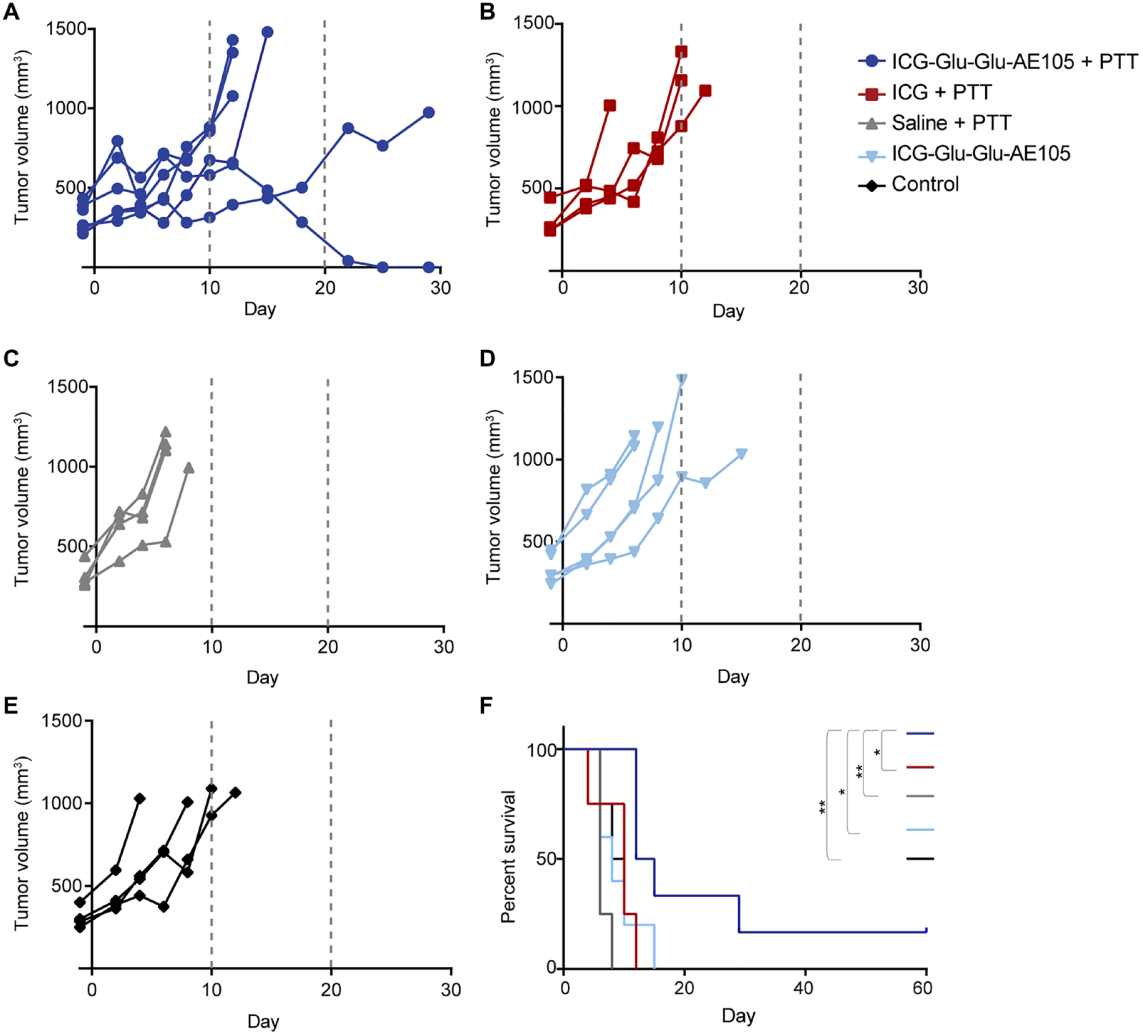
Tumor growth and survival after ICG-Glu-Glu-AE105-based PTT. (**A**–**E**) Growth curves from the ICG-Glu-Glu-AE105 + PTT (*n* = 6), ICG + PTT (*n* = 4), Saline + PTT (*n* = 4), ICG-Glu-Glu-AE105 (*n* = 5) and Control (*n* = 4) groups respectively. Curves stopped on day 29 as only one ICG-Glu-Glu-AE105 +PTT mouse was left. (**F**) Survival curves for the different groups. When comparing survival curves between the ICG-Glu-Glu-AE105 + PTT group and the different control groups, ^*^denotes *p* value < 0.05 and ^**^denotes *p* value < 0.01.

The high temperatures reached by the ICG-Glu-Glu-AE105 + PTT group correlate with the delay in tumor growth that the group presented when compared to all the other groups (Figure 4A–4E). Accordingly, survival was improved, and one mouse even experienced complete tumor disappearance three weeks after therapy with no recurrence up until day 60 after PTT, when the study was terminated (Figure 4F). No significant differences in tumor growth or survival were detected between the different control groups. The median survival was 13.5 days for ICG-Glu-Glu-AE105 group and 10 days for the ICG group, and the hazard ratio (HR) of ICG-Glu-Glu-AE105 vs. ICG group was 0.28 (95% CI = 0.051-1.5; *p* = 0.0115). As for all the other groups, median survival was 6 days for the saline group (HR for ICG-Glu-Glu-AE105 vs. saline group of 0.22, 95% CI = 0.035–1.37; *p* = 0.0013), 8 days for the sham group (HR for ICG-Glu-Glu-AE105 vs. sham group of 0.32, 95% CI = 0.075–1.36; *p* = 0.0265) and 9 days for the control group (HR for ICG-Glu-Glu-AE105 vs. control group of 0.26, 95% CI = 0.046-1.47; *p* = 0.0097).

**Figure 5:**
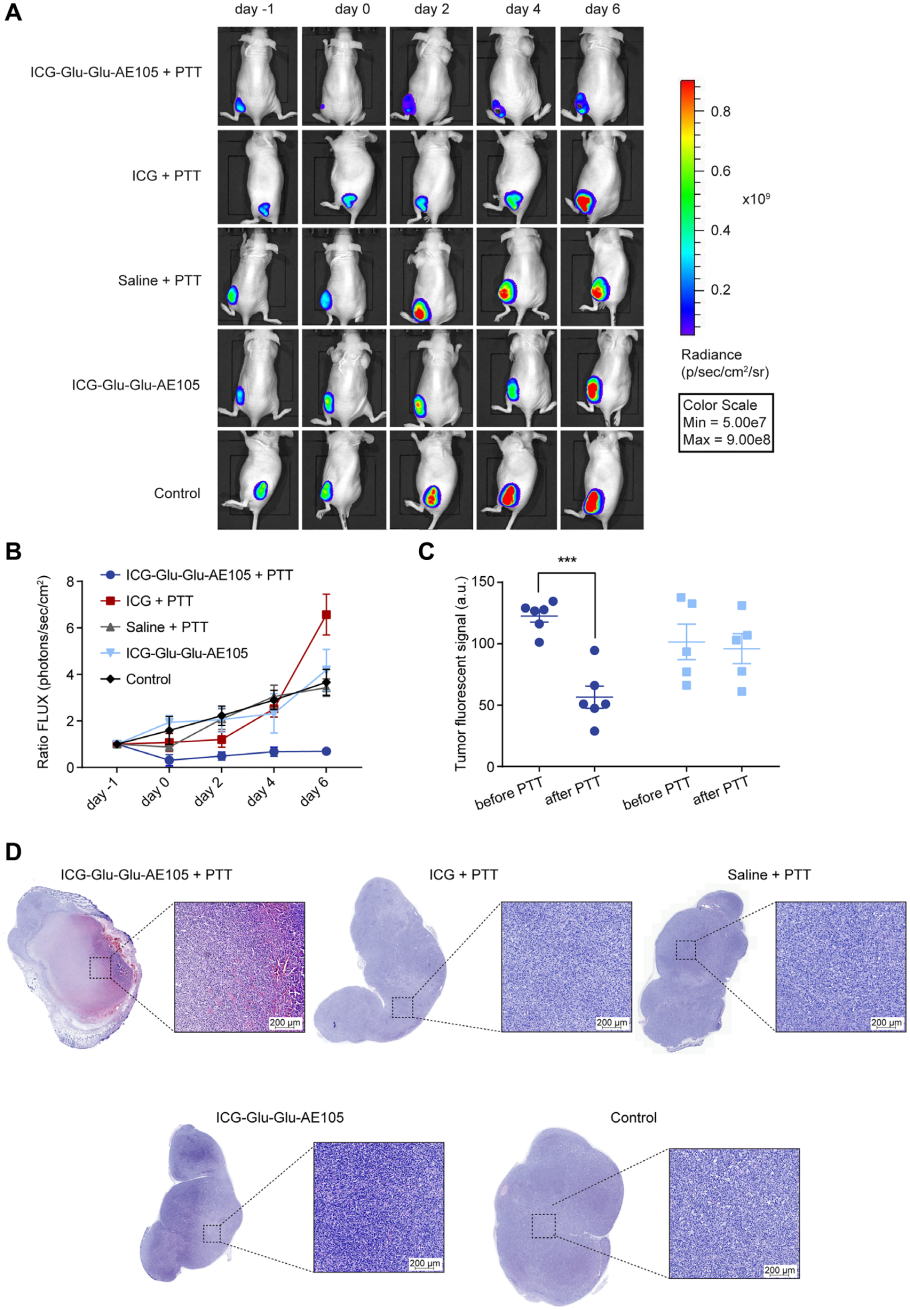
Bioluminescence signal after PTT. (**A**) Representative images of bioluminescence scans at different timepoints for all the groups; ICG-Glu-Glu-AE105 (46 nmol) + PTT (*n* = 6), ICG (46 nmol) + PTT (*n* = 4), Saline + PTT (*n* = 4), ICG-Glu-Glu-AE105 (46 nmol, *n* = 5) and Control (*n* = 4). (**B**) Bioluminescent signal expressed as ratio FLUX (day x/day-1) for the different groups. Data shown from day -1 to day 6, whereafter *n* < 3 in some groups. (**C**) Differences in tumor fluorescent signal detected by Fluobeam for ICG-Glu-Glu-AE105 + PTT and ICG-Glu-Glu-AE105 groups. ^***^denotes *p* < 0.001. Data shown as mean ± SEM. (**D**) H&E staining of tumor tissue 2 days after PTT. ICG-Glu-Glu-AE105 + PTT tumors showed tissue disruption and hemorrhage, meanwhile all the other groups presented normal viable tumor tissue. *n* = 2 per group.

In line with these results, the bioluminescence imaging after treatment showed a reduced signal for the ICG-Glu-Glu-AE105 + PTT group due to tumor death caused by PTT (Figure 5A). This low signal was maintained throughout the study and stayed significantly lower than in all the other groups (Figure 5B).

Fluorescence scans were performed to confirm the ICG degradation in the ICG-Glu-Glu-AE105 molecules after irradiation at high laser intensity, as already observed *in vitro*. As expected, the fluorescence signal in the tumor for the ICG-Glu-Glu-AE105 + PTT group was reduced significantly after PTT, which did not happen for non-irradiated ICG-Glu-Glu-AE105-bearing tumors (Figure 5C).

Finally, tumors from mice receiving the different treatments (*n* = 2) were resected at day 2 (after PTT) and H&E stained to confirm changes in tumor morphology due to treatment *ex vivo* (Figure 5D). As expected, ICG-Glu-Glu-AE105 + PTT mice showed a high degree of cell death when compared to all the other groups, which presented completely viable tissue throughout the entire tumor, thereby not showing any treatment effect.

## DISCUSSION

Indocyanine green (ICG), an FDA approved fluorophore that has been widely used for NIR imaging, could potentially also be applied as a photothermal agent. However, the use of ICG for PTT has been limited due to its rapid clearance from the body and non-specific tumor targeting [12, 21]. To overcome these limitations, new strategies based on novel ICG-conjugated nanoparticles have been developed in order to increase the ICG concentration in the tumor. For instance, ICG-loaded self-assembled hyaluronic acid nanoparticles were able to enhance intraoperative contrast and increase the number of complete breast tumor resections [22, 23]. Additionally, liposomal formulations of ICG were able to maximize its photothermal effect, and allowed for photothermal treatment monitored by NIR fluorescence-based imaging [24]. However, many of these platforms rely on passive accumulation, e.g., through enhanced permeability and retention (EPR), to reach the tumor, and many inorganic nanoparticle-based systems can have long retention times and limited body clearance of potentially toxic nanoparticle components [25, 26].

uPAR is a receptor known to be overexpressed in multiple tumor types and is therefore a promising target for anti-cancer therapy [13, 16, 27]. AE105 is a small peptide that binds to uPAR and has previously been used for imaging and targeted therapy [18, 28, 29]. The strong uPAR expression at the invasive tumor front and surrounding stroma makes it highly interesting for guided tumor resection, and more recently the fluorescent probe composed of ICG and AE105, ICG-Glu-Glu-AE105, has been applied for fluorescence-guided surgery [30, 31]. In studies using mice bearing xenograft tumors, a high tumor-to-background ratio of ICG-Glu-Glu-AE105 was observed, and the fluorescent probe showed its potential to improve surgical outcome [16, 31, 32]. Based on these findings, a first-in-humans clinical trial using ICG-Glu-Glu-AE105 for image-guided surgery in brain cancer patients was recently initiated (EudraCT: 2020-003089-38). In addition to guiding surgery, this tumor-delineating expression profile also makes uPAR interesting for targeted ablation. Therefore, we wanted to test the ability of ICG-Glu-Glu-AE105 as a photothermal agent. We found a high increase in tumor temperature when ICG-Glu-Glu-AE105-based PTT was performed in a uPAR-expressing mouse tumor model, which also resulted in a delay in tumor growth. One mouse even experienced complete tumor disappearance, sustained until day 60 when the study was terminated. In addition, this effect was achieved by treating animals as early as four hours after injection of the probe, and the ICG-Glu-Glu-AE105 uptake remained stable in the tumor for a longer period of time than when injecting unconjugated ICG. Much higher doses of the unconjugated form would be needed in order get a comparable outcome [33]. The effect of the treatment on the group receiving ICG-Glu-Glu-AE105-based PTT also resulted in a decrease in bioluminescent signal due to the laser-induced cell death, and a significant degree of tissue damage was observed through H&E staining. In contrast, we found no effect on tumor growth in any of the control groups. This also applied for the group of mice treated with unconjugated ICG and PTT, and indicates a potential advantage of applying the targeted approach for ICG delivery to the tumor.

For proof-of-concept, the experiments were performed in subcutaneous tumors with a volume of around 300 mm^3^. The results presented here show the feasibility of ICG-Glu-Glu-AE105 as a probe for PTT, a potential add-on to its applicability as a promising imaging agent for fluorescence-guided surgery. Further studies are needed to validate the probe for this application, but it is our hope that ICG-Glu-Glu-AE105-based PTT could serve as an adjuvant method applied during fluorescence-guided surgery to improve clinical outcome.

## MATERIALS AND METHODS

### Materials

The peptide AE105 was conjugated to ICG by ABX (Radeberg, Germany) [16, 34].

### *In vitro* experiments

The ability of ICG to generate high temperatures when irradiated with NIR light was measured *in vitro*. Different concentrations (228, 46, 23 and 12 nmol) of unconjugated ICG and ICG-Glu-Glu-AE105 in solution (2-hydroxypropyl)-β-cyclodextrin, HβC, in water and 2% DMSO) were placed in a plastic cuvette under an 807-nm laser beam (beam diameter of ~1 cm). The samples were irradiated for five minutes at 2 W/cm^2^ and the temperature was monitored real-time with a thermal camera (FLIR T-440 camera), taking images every 30 seconds. The images were analyzed using the FLIR tools software.

### Cell line and animal model

The animal experiments were performed under a protocol approved by the Danish Animal Welfare Council, Ministry of Justice (2016-15-0201-00920). For the *in vivo* studies, U-87 MG.luc2 cells were cultured in Dulbecco’s modified eagle medium supplemented with 10% fetal bovine serum and 1% penicillin-streptomycin (Thermo Fisher Scientific) at 37°C and in 5% CO_2_. When cells reached ~70% confluence, they were harvested and 2–3 × 10^6^ cells in 100 μl of PBS were inoculated into the left flank of NMRI nude female mice (Janvier Labs, France). Animals were left to grow tumors until they reached ~300 mm^3^. From then on, tumors were measured every other day with the use of a caliper, and tumor volume calculated through the formula: (length × width^2^) × 0.5. When tumors reached ~1,000 mm^3^, animals were euthanized.

### Flow cytometry

Flow cytometry was performed to confirm uPAR expression in U-87 MG.luc2 cells. When cells reached ~70% confluence, they were harvested with non-enzymatic cell dissociation buffer (Thermo Scientific) and washed in PBS buffer. Afterwards, cells were stained with Viability Dye (eBioscience, California, USA) and incubated with FC block in flow cytometry staining buffer (0.5% BSA, 0.1% Sodiumazide and 2 mM EDTA in PBS) to decrease non-specific binding. Then, they were stained with either a PE anti-human CD87 (uPAR) antibody (#555768, BD Biosciences, California, USA) or a PE IgG1 isotype control (#555749, BD Biosciences) for 30 min at 4°C. The samples were run in a BD LSRFortessa™ cell analyzer (BD Biosciences) and the results analyzed within the FlowJo software v.10.6.

### ICG-Glu-Glu-AE105 accumulation *in vivo*

Animals were injected intravenously with either ICG-Glu-Glu-AE105 (23 or 46 nmol; 0.25 mg/mL and 0.5 mg/mL respectively) or ICG alone (46 nmol; 0.18 mg/mL) in 200 μl of 0.2 g/mL HβC in water and 2% DMSO. The mice (*n* = 3–5 per group) were then imaged with the Fluobeam^®^800 NIR-camera (Fluoptics, Grenoble, France) at different time points (1, 2, 3, 4, 6, 8, 15 and 24 h). For this, they were placed below the optical beam while kept under anesthesia by breathing 4% sevoflurane. Images were analyzed within the ImageJ software, where regions of interest (ROIs) were manually drawn on the tumors and extracted as fluorescence signal (arbitrary units).

### *In vivo* PTT

When tumors reached 300 mm^3^, mice were divided into 5 groups: ICG-Glu-Glu-AE105 + PTT group (mice injected with 46 nmol ICG-Glu-Glu-AE105 four hours before PTT, *n* = 6); ICG + PTT group (mice injected with 46 nmol of unconjugated ICG four hours before PTT, *n* = 4); Saline + PTT group (mice injected with saline four hours before PTT, *n* = 4); ICG-Glu-Glu-AE105 group (mice injected with 46 nmol ICG-Glu-Glu-AE105 and no PTT, *n* = 5) and a control group (no injection, no PTT, *n* = 4). Animals were given analgesia (buprenorphine, 0.3 mg/ml) right before the laser treatment and every 6 to 8 hours until deemed necessary. For PTT, animals were anesthetized by breathing 4% sevoflurane, placed on the treatment platform and had the tumors irradiated for five minutes at a laser intensity of 2 W/cm^2^. The tumors were swabbed with glycerol, an index-matching agent, prior to the laser treatment to facilitate light penetration [35–38]. The temperatures reached on the tumor surface were recorded with a thermal camera, as described for the *in vitro* experiments. Animals that did not undergo PTT (control and sham groups) were placed on the treatment platform to mimic the process.

### Bioluminescence and fluorescence imaging

Animals were scanned for bioluminescence using the IVIS Lumina XR (Caliper life Sciences, Hopkinton, CA, USA) a day before PTT (day -1), on PTT day (day 0) and afterwards every other day until endpoints were reached. For this, mice were injected with luciferin intraperitoneally (5 μl/mg of body weight, at a concentration of 150 mg/mL) ten minutes before the scan. Animals were then anesthetized and placed in the prone position. The bioluminescent signal was quantified within the acquisition software Living Image (Caliper Life Sciences, Hopkinton, CA, USA) by drawing ROIs on the tumors and obtaining the photon flux (photons/s/cm^2^).

The fluorescence scans were performed to detect ICG signal before and after PTT using the Fluobeam^®^800 NIR-camera as described for the accumulation study.

### Histological analysis

Tumors were resected from *n* = 2 mice per group two days after PTT. Tumors were fixated in formaldehyde overnight, and afterwards conserved in ethanol for their later embedding in paraffin. To perform the histology experiments, tumors were cut into 4 μm slices in a microtome (Thermo Scientific Rotary Microtome Microm HM355S). The tissue slides were left to dry for at least 1.5 hours, and then heated first at 40 and afterwards at 60°C. Following, slides were submerged in HistoClear solution to achieve deparaffinization and rehydrated in a series of ethanol to water. For H&E staining, the tissue slides were stained in hematoxylin for five minutes, rinsed and stained with eosin for three minutes. Sections were scanned on Zeiss Axio Scan.Z1.

### Statistics and data analysis

The Kaplan-Meier method was used to create the survival curves, and they were compared through the log-rank test. The fluorescence emitted by ICG before and after PTT was compared using a paired *t*-test. The data was plotted in Prism7 and shown as mean ± SEM (standard error of the mean).

## Abbreviations

PTT: photothermal therapy
ICG: indocyanine green
NIR: near-infrared
uPAR: urokinase plasminogen activator receptor
